# Regulatory T Cells in Human Lymphatic Filariasis: Stronger Functional Activity in Microfilaremics

**DOI:** 10.1371/journal.pntd.0001655

**Published:** 2012-05-29

**Authors:** Linda J. Wammes, Firdaus Hamid, Aprilianto E. Wiria, Heri Wibowo, Erliyani Sartono, Rick M. Maizels, Hermelijn H. Smits, Taniawati Supali, Maria Yazdanbakhsh

**Affiliations:** 1 Department of Parasitology, Leiden University Medical Center, Leiden, The Netherlands; 2 Department of Parasitology, University of Indonesia, Jakarta, Indonesia; 3 Department of Microbiology, Hasanuddin University, Makassar, Indonesia; 4 Institute of Immunology and Infection Research, University of Edinburgh, Edinburgh, United Kingdom; Uniformed Services University, United States of America

## Abstract

Infection with filarial parasites is associated with T cell hyporesponsiveness, which is thought to be partly mediated by their ability to induce regulatory T cells (Tregs) during human infections. This study investigates the functional capacity of Tregs from different groups of filarial patients to suppress filaria-specific immune responses during human filariasis. Microfilaremic (MF), chronic pathology (CP) and uninfected endemic normal (EN) individuals were selected in an area endemic for *Brugia timori* in Flores island, Indonesia. PBMC were isolated, CD4CD25^hi^ cells were magnetically depleted and *in vitro* cytokine production and proliferation in response to *B. malayi* adult worm antigen (BmA) were determined in total and Treg-depleted PBMC. In MF subjects BmA-specific T and B lymphocyte proliferation as well as IFN-gamma, IL-13 and IL-17 responses were lower compared to EN and CP groups. Depletion of Tregs restored T cell as well as B cell proliferation in MF-positives, while proliferative responses in the other groups were not enhanced. BmA-induced IL-13 production was increased after Treg removal in MF-positives only. Thus, filaria-associated Tregs were demonstrated to be functional in suppressing proliferation and possibly Th2 cytokine responses to BmA. These suppressive effects were only observed in the MF group and not in EN or CP. These findings may be important when considering strategies for filarial treatment and the targeted prevention of filaria-induced lymphedema.

## Author Summary

Lymphatic filariasis is a neglected disease still prominent in low-resource settings and is very disabling when it progresses to chronic pathology caused by lymphedema. Until now, studies on the contribution of Tregs to lymphocyte hyporesponsiveness in human filariasis have focused on frequency and phenotypic characteristics of these cells. We have looked at the functional consequence of the presence of Tregs in filaria-specific immune responses during different stages of human lymphatic filariasis. Proliferation of not only T cells, but also B cells, was decreased in patients with microfilaremia compared to uninfected individuals and chronic pathology (lymphedema) patients. The suppressed lymphocyte proliferative responses were increased after *in vitro* removal of Tregs in the microfilaria-positive group only, indicating the presence of filaria-specific functional Tregs in microfilaremic patients which are not as active in subjects with chronic pathology or without infection. Th2 cytokine responses were specifically enhanced in microfilaremics as well after Treg depletion, suggesting Treg-associated suppression of filaria-specific Th2 responses. Taken together, filaria-specific Treg contribute to immune modulation during microfilaremia and might need to be considered in therapeutic strategies to prevent chronic pathology induced by filarial infection.

## Introduction

Lymphatic filariasis (LF), caused by nematodes *Wuchereria bancrofti*, *Brugia malayi* and *B. timori*, affects around 120 million people worldwide and additionally 2 billion people are at risk in endemic areas [1]. Although not life-threatening, chronic manifestation of disease causes major disabilities and deformities, especially in areas with minimal access to health care facilities. Indonesia is one of the endemic countries in the South-East Asia region and accounts for the second highest burden of LF in the world. All three filarial parasites are prevalent in the archipelago and efforts are being made to control the disease in various areas (Global Programme to Eliminate Lymphatic Filariasis) [2], [3].

Helminths such as filarial parasites have been shown to induce immune modulation, resulting in T cell hyporesponsiveness and failure to expel parasites [4]. Initially a phase of immune activation and proinflammatory cytokine responses is induced by the larval stages of filarial parasites [5]. However in patent infection, with circulating microfilariae (MF) and/or filarial antigens, decreased proliferative responses and increased anti-inflammatory cytokines, such as IL-10 and TGF-β, reflect a state of immune hyporesponsiveness [6]. At the transcriptional level, it has been shown that in infected subjects both Th1 and Th2 pathways are downmodulated by the enhanced expression of molecules such as FOXP3, CTLA-4 and TGF-β involved in regulatory networks [7]. In patients with chronic pathology this seems to be reversed; in PBMC from these patients enhanced inflammatory Th1 and Th17 responses as well as decreased levels of mRNA for different Treg markers were observed as compared to asymptomatic infected individuals [8].

The suppressive capacities of Tregs have been implicated in many infectious diseases, including filariasis. Induction of Tregs by pathogens is regarded as one of the mechanisms to evade the human immune system [9]. A recent report demonstrated that in animal models, early recruitment of Tregs affects the course of the immune response that leads to the development of chronic filariasis, indicating that Tregs are important regulators of the overall immune response to filarial nematodes in mice [10]. In human filariasis, different Treg subsets have been the focus of recent studies in different age groups and different clinical categories. While in India, higher frequencies of regulatory T cell markers were found in asymptomatic microfilaremics compared with chronic pathology patients, a recent study in Mali reported higher frequencies of Tregs (CD4^+^CD25^+^FOXP3^+^CD127^−^) in MF or circulating filaria antigen-positive versus uninfected adolescents, but also suggested a more prominent regulatory role for IL-10 producing, so-called adaptive Tregs (CD4^+^CD25^−^) cells [8], [11].

Altogether, in previous studies of regulatory networks in human filariasis phenotypes of participating lymphocyte subsets and key regulatory molecules have been investigated, whereas the functional capacity of Tregs remained largely unknown. In this study we aimed to explore the immune regulatory activity in different disease states of microfilaremia (MF), chronic pathology (CP) presented as elephantiasis and uninfected endemic normals (EN) as controls in a population living in an area endemic for *B. timori* in Indonesia. By *in vitro* depletion assays we determined the effect of Tregs on filaria-specific T and B cell proliferation and cytokine production.

## Methods

### Study population and parasitological diagnostics

In Sikka district, Flores, east Indonesia, an area endemic for *B. timori* was identified. Study participants were recruited from surrounding villages, written informed consent was obtained and night blood samples were collected to determine microfilaremia. Morning venous blood samples were collected from 24 MF-negative asymptomatic endemic normals (EN), 24 MF-positive asymptomatic individuals (MF) and 26 MF-negative chronic pathology (uni- or bilateral elephantiasis) patients (CP). 1 ml of blood was used for filtration to quantify mf load and thick blood smears were screened for the presence of malaria parasites. The study was approved by the Committee of Medical Research Ethics of the University of Indonesia.

### Cell isolation and Treg depletion

Peripheral blood mononuclear cells (PBMC) were obtained by gradient centrifugation of heparinized venous blood over Ficoll. Based on sufficient numbers of PBMC, of 69 individuals (23 in each group) CD4^+^CD25^hi^ T cells were isolated by magnetic cell sorting (MACS) using the CD4^+^CD25^+^ Regulatory T Cell Isolation Kit (Miltenyi Biotec GmBH, Bergisch Gladbach, Germany); details have been described previously [12]. The CD4^+^CD25^hi^ -depleted PBMC were compared with PBMC which were treated in an identical manner, however to which the eluted CD4^+^CD25^hi^ cells were added back to (this is referred to as “mock-depleted”).

### PBMC stimulation assay for proliferation and cytokine production

The green-fluorescent dye carboxyfluorescein succinimidyl ester (CFSE; Sigma-Aldrich, CA, USA) was used to monitor proliferation. CFSE is divided over daughter cells upon cell division and this can subsequently be tracked by decreasing fluorescence intensity. After labeling with 2 µM CFSE, mock- and CD4^+^CD25^hi^ -depleted PBMC were cultured in RPMI 1640 (Gibco, Invitrogen, Carlsbad, CA, U SA) supplemented with 10% FCS (Greiner Bio-One GmbH, Frickenhausen, Germany) with or without *B. malayi* adult worm antigen (BmA, 10 µg/ml). After 96 h cell supernatants were collected and cells were fixed in 2% formaldehyde (Sigma-Aldrich), after which all samples were preserved at −20°C first, then at −80°C.

### Flowcytometry

After thawing, the CFSE-positive cells were labeled with fluorochrome-conjugated anti-CD3, anti-CD4, anti-CD25 (BD Biosciences, Franklin Lakes, NJ, USA) and anti-CD19 (biotinylated antibody from eBioscience Inc., San Diego, CA, USA; streptavidin-Qdot525 from Invitrogen) antibodies, acquired on a FACSCanto II machine (BD Biosciences) and analyzed with FlowJo software (Treestar Inc., Ashland, OR, USA). Proliferation of effector T cells was determined in a FlowJo Proliferation application by calculation of the fraction of cells from the starting population that had divided, within the CD3^+^CD4^+^CD25^+^ T cell and CD3^−^CD19^+^ B cell subsets. Since background levels of cell proliferation were high, spontaneous proliferation was subtracted from BmA-stimulated values to compare proliferative responses in the three study groups.

### Cytokine multiplex analysis

Cytokine production was assessed using the Multiplex Bead Immunoassay for interferon-gamma (IFN-γ), interleukin (IL)-13, IL-17 and IL-10 according to the protocol supplied by the manufacturer (Biosource, Invitrogen, Carlsbad, CA, USA). Samples were acquired with Luminex 100™ xMAP technology (Luminex Corp., Austin, TX, USA). Half the detection limit supplied by the manufacturer was used for values below detection limit and the values above upper detection limit were given the upper limit value. The cytokine data were not normally distributed and therefore are presented as raw unmanipulated data. Thus, there was no subtraction of or division over unstimulated samples, but data are shown separately as medium-stimulated or antigen-stimulated cytokines.

### Data analysis

Statistical analysis was performed in SPSS 16.0. Not-normally distributed values (cytokine levels in supernatants) were log-transformed. Both age and sex were incorporated into univariate analysis to compare different infection and clinical groups. Resulting adjusted means were anti-log-transformed when needed. For mock- versus Treg-depleted samples, paired analysis was done using paired t-test or Wilcoxon Signed Ranks Test. In the multiplex cytokine analysis Bonferroni correction was taken into account where applicable, by multiplying the p-values by the number of non-correlated measurements.

## Results

### Study population and parasitological examination

Individuals from an area endemic for lymphatic filariasis in the north of Flores, Indonesia, were recruited for a night blood survey. Based on the microfilaremic status and sufficient number of PBMC, 23 MF-negative asymptomatic endemic normals (EN), 23 MF-positives (MF) and 23 chronic pathology (CP) patients were included for immunological studies. Microscopic *Plasmodium* spp. parasitemia was found in 2 CP patients, but had no effect on the analyses shown here. The characteristics of the study population are summarized in Table 1. Age was significantly higher in the CP group (medians 42, 46 and 54 years for EN, MF and CP respectively; p = 0.038), while male to female ratio was lower in the CP group (percentage male 48%, 65% and 22% in EN, MF and CP respectively; p = 0.012). Because of these differences, comparisons between groups were adjusted for age and sex. The lymphocyte count (PBMC/ml blood) was similar in the three groups (medians 1.09, 0.97 and 0.92 for EN, MF and CP respectively), as well as the frequencies of T and B cells (data not shown).

**Table 1:** Study population characteristics.

	*endemic uninfected (EN)*	*microfilaremic (MF)*	*chronic pathology (CP)*	*p*	*total*
**N**	23	23	23		69
**age**	42	46	54	0.038	46
(median [range])	[19–67]	[14–72]	[20–76]		[14–76]
**sex**	11/12	15/8	5/18	0.012	31/38
(M/F)					
**malaria parasitemia**	0	0	1,1	ns	2
(n of *Pf*, *Pv*)*					
**PBMC/ml** (·106)	1.09	0.97	0.92	ns	0.98
(median [range])	[0.6–2.3]	[0.7–5.6]	[0.7–1.6]		[0.6–5.6]

*: *Pf Plasmodium falciparum; Pv Plasmodium vivax.*

### Filaria-specific proliferative responses of T and B cells are suppressed in microfilaremics

**Figure 1:**
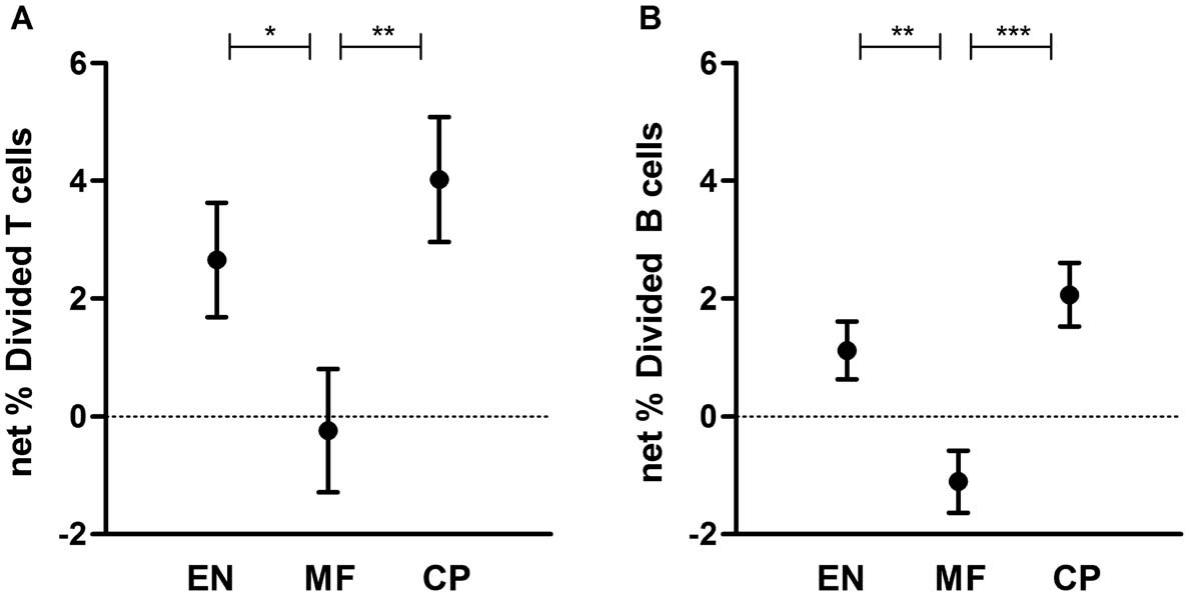
Suppressed T cell and B cell proliferative response to filaria antigen in microfilaremics. CFSE-labeled PBMC from uninfected endemic normals (EN), microfilaremic (MF) and chronic pathology (CP) subjects were stimulated with *Brugia malayi* adult worm antigen (BmA). After 4 days of culture cells were fixed, cryopreserved and after thawing CFSE division was analyzed by flow cytometry. Depicted are means and standard errors of net % divided subsets of CD4^+^CD25^+^ T cells (A) and CD19^+^ B cells (B), adjusted for age and sex. * p≤0.05 **p≤.01 ***p≤.001.

To analyze suppression of lymphocyte proliferation during filarial infection, cell proliferation to filarial antigen was determined by CFSE dilution in PBMC. Divided cell subsets were measured in activated T (CD4^+^CD25^+^) and in B (CD19^+^) cell populations. Net T cell proliferation was lower in the MF group, which was mainly caused by high background proliferation in unstimulated condition (response to medium, Figure 1A; age- and sex-adjusted means 2.66%, −0.236%, 4.02% divided in EN, MF and CP respectively; p = 0.043 for EN vs. MF, p = 0.010 for MF vs. CP). Also B cell proliferation was lower in MF, shown in figure 1B (adjusted means 1.13%, −1.11%, 2.07% divided for EN, MF and CP respectively; p = 0.002 for EN vs. MF and p = 0.0002 for MF vs. CP).

### Lower filaria-specific Th1, Th2 and Th17 cytokine responses in MF

**Figure 2:**
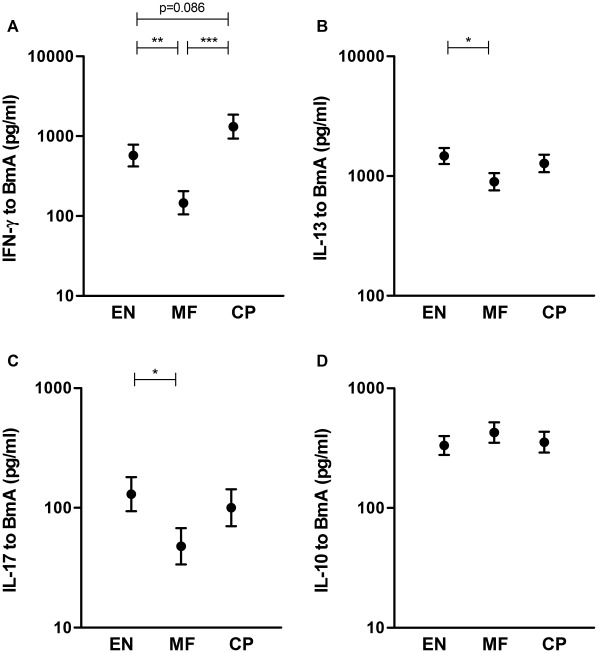
Altered filaria-specific cytokine production in different study groups. PBMC from uninfected endemic normals (EN), microfilaremic (MF) and chronic pathology (CP) subjects were stimulated with BmA. After 4 days of culture supernatants were harvested and assessed for IFN-γ (A), IL-13 (B), IL-17 (C) and IL-10 (D) production. Plotted values are age- and sex-adjusted means and standard errors; *p≤.05 **p≤.01 ***p≤.001, p-values between 0.05 and 0.10 are indicated.

To assess the modulation of differentiated T helper cell subsets by filarial infection, hallmark cytokines for Th1 (IFN-γ), Th2 (IL-13), Th17 (IL-17) and regulatory (IL-10) responses were assessed in culture supernatants from cells stimulated with BmA (Figure 2). IFN-γ production was lower in the MF group than in EN or CP (adjusted means 573, 146 and 1318 pg/ml in EN, MF and CP respectively; p = 0.004 for EN vs. MF, p = 0.00007 for MF vs. CP). Both IL-17 and IL-13 levels were decreased in the MF group compared to EN, however were not different from CP (IL-17 adjusted means 130, 48 and 100 pg/ml; p = 0.037 for EN vs. MF; IL-13 adjusted means 1472, 895 and 1276 pg/ml in EN, MF and CP respectively; p = 0.029 for EN vs. MF). IL-10 production was similar in all three groups (adjusted means 333, 427 and 355 pg/ml in EN, MF and CP respectively). Spontaneous production of these cytokines was not significantly different between the groups and it was noted that IFN-γ and IL-17 levels in BmA-stimulated PBMC supernatants were hardly above spontaneous (unstimulated) production, particularly in the MF group (Figure S1). After applying correction for multiple analyses, only IFN-γ levels were significantly lower in microfilaremic individuals.

### Similar Treg depletion in EN, MF and CP groups

**Figure 3:**
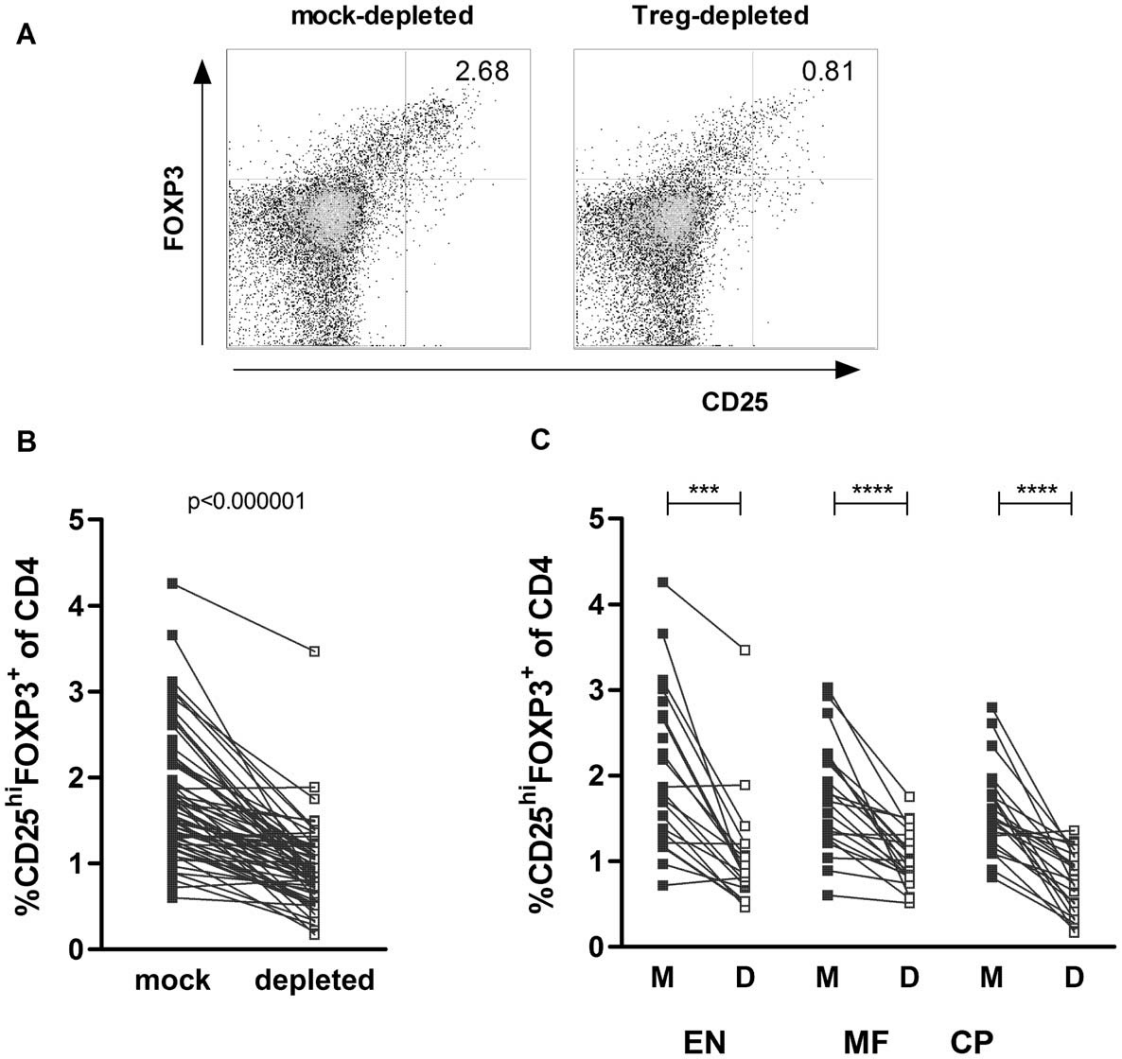
Efficient Treg depletion for all infection groups. CD4^+^CD25^hi^ T cells were isolated by magnetic bead separation. Treg frequencies were defined as percentages of CD25^hi^FOXP3^+^ cells from total CD4^+^ fractions for mock- and CD4^+^CD25^hi^ cell -depleted PBMC that were cultured for 4 days in medium (representative example in A). Treg frequency of mock (M) and depleted (D) PBMC is shown for all donors (B) as well as for the different infection groups (C). Lines represent data points from one individual, data were analyzed by non-parametric paired tests; ***p≤0.001 ****p≤0.0001 or p-value as indicated.

To assess the functional contribution of Tregs to *in vitro* immune responses, we performed magnetic depletion of CD4^+^CD25^hi^ cells. By flowcytometry mock- and Treg-depleted PBMC were assessed for expression of CD25 and FOXP3 on CD4 T cells, of which a representative example is shown in Figure 3A. Treg frequencies decreased in most cases (Figure 3B), which was highly significant and similar in all three clinical groups (Figure 3C; p = 1.7•10^−4^ for EN, p = 2.7•10^−5^ for MF, p = 3.1•10^−5^ for CP). Geometric mean of CD25^hi^FOXP3^+^ cell percentages of CD4 cells decreased from 1.69% to 0.83% after depletion (mean extent of depletion was 46.5%). For 5 donors, 4 in EN and 1 in CP group, Treg frequency either could not be assessed or did not decrease after depletion, therefore these patients were excluded for further analysis.

### Depletion of Tregs enhances filaria-specific lymphocyte proliferation

**Figure 4:**
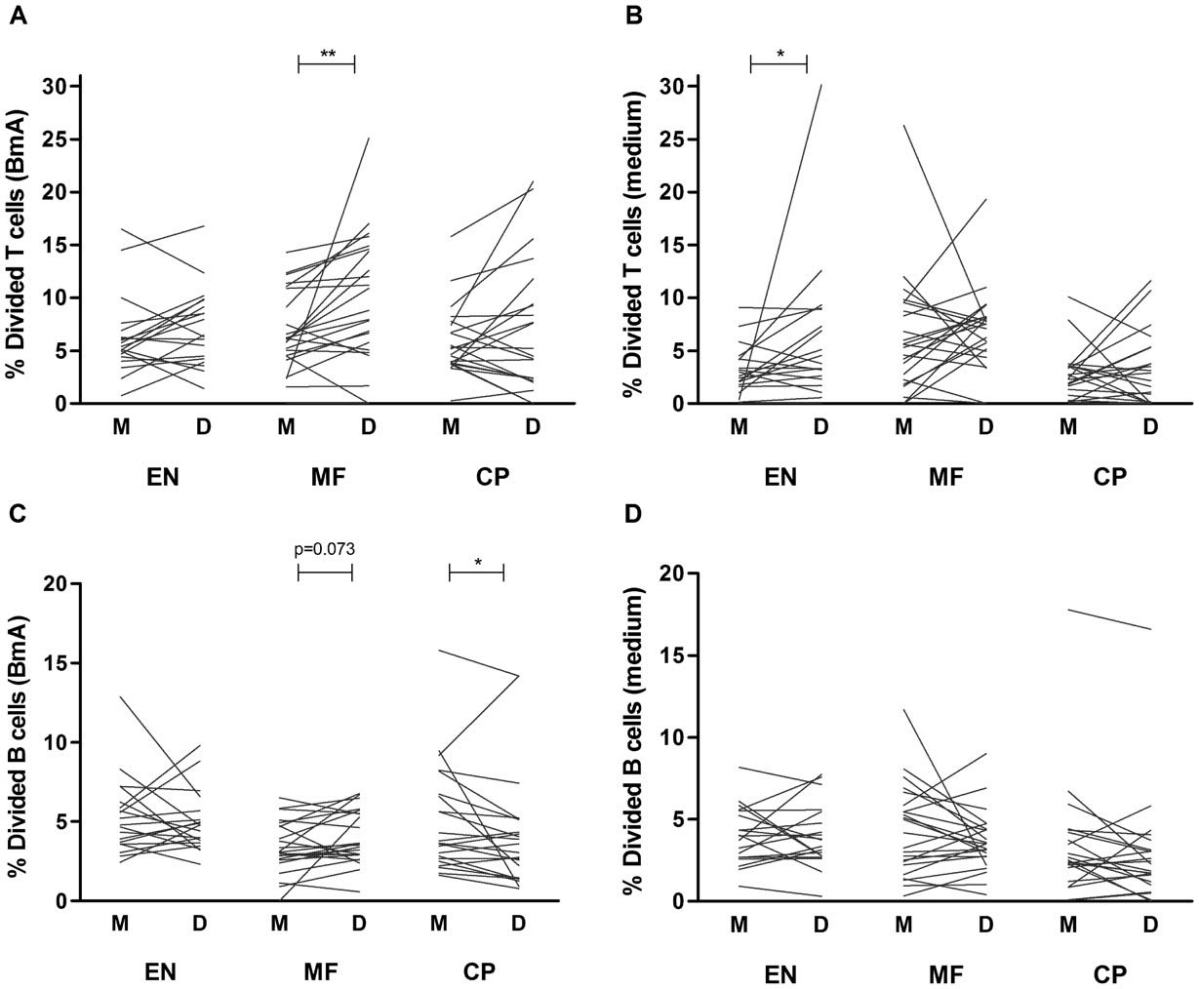
Suppressed lymphocyte proliferation is restored after Treg depletion in microfilaremics. Divided cell populations were assessed in mock- (M) and Treg-depleted (D) PBMC from EN, MF and CP subjects. CFSE dilution was analyzed for CD4^+^CD25^+^ T cells (A–B) and CD19^+^ B cells (C–D). Cultures were stimulated with BmA (left panel; A&C) or left unstimulated (cultured with medium) (right panel; B&D). Plotted lines represent data points from single individuals; tested by Wilcoxon signed rank test, *p≤.05 **p≤.01, p-values between 0.05 and 0.10 are indicated.

To evaluate the influence of Tregs on BmA-specific T and B cell proliferation, we analyzed CFSE dilution in CD4^+^CD25^+^ and CD19^+^ cell subsets before and after Treg depletion. Here we present unadjusted proliferative responses to BmA and medium separately. For CD4^+^CD25^+^ effector T cells we observed an increase in proliferation to BmA in the MF group after removal of Tregs, whereas proliferative responses did not change significantly in EN or CP groups (Figure 4A; p = 0.004 for MF). Treg depletion did not enhance spontaneous proliferation (medium condition) in MF-positives, however spontaneous responses were increased in uninfected individuals (Figure 4B, p = 0.04 for EN). Interestingly, B cell proliferative responses in response to BmA were also enhanced in Treg-depleted conditions for MF patients, although this fell short of statistical significance (Figure 4C; p = 0.07 for MF). In contrast, after Treg depletion B cells proliferated to a lesser extent in CP patients (Figure 4C; p = 0.01 for CP). Unstimulated B cell proliferative responses were not influenced by Treg removal (Figure 4D). To check whether Treg depletion completely restored lymphocyte proliferative responses in the MF group to levels seen in the other groups, we compared age- and sex-adjusted net proliferative responses of T and B cells to BmA in Treg-depleted conditions. Although responses in the CP group remained high for both T and B cells, T and B cell proliferation in the MF group was no longer different from EN individuals (Figure S2).

### Depletion of Tregs enhances filaria-specific Th2 responses

**Figure 5:**
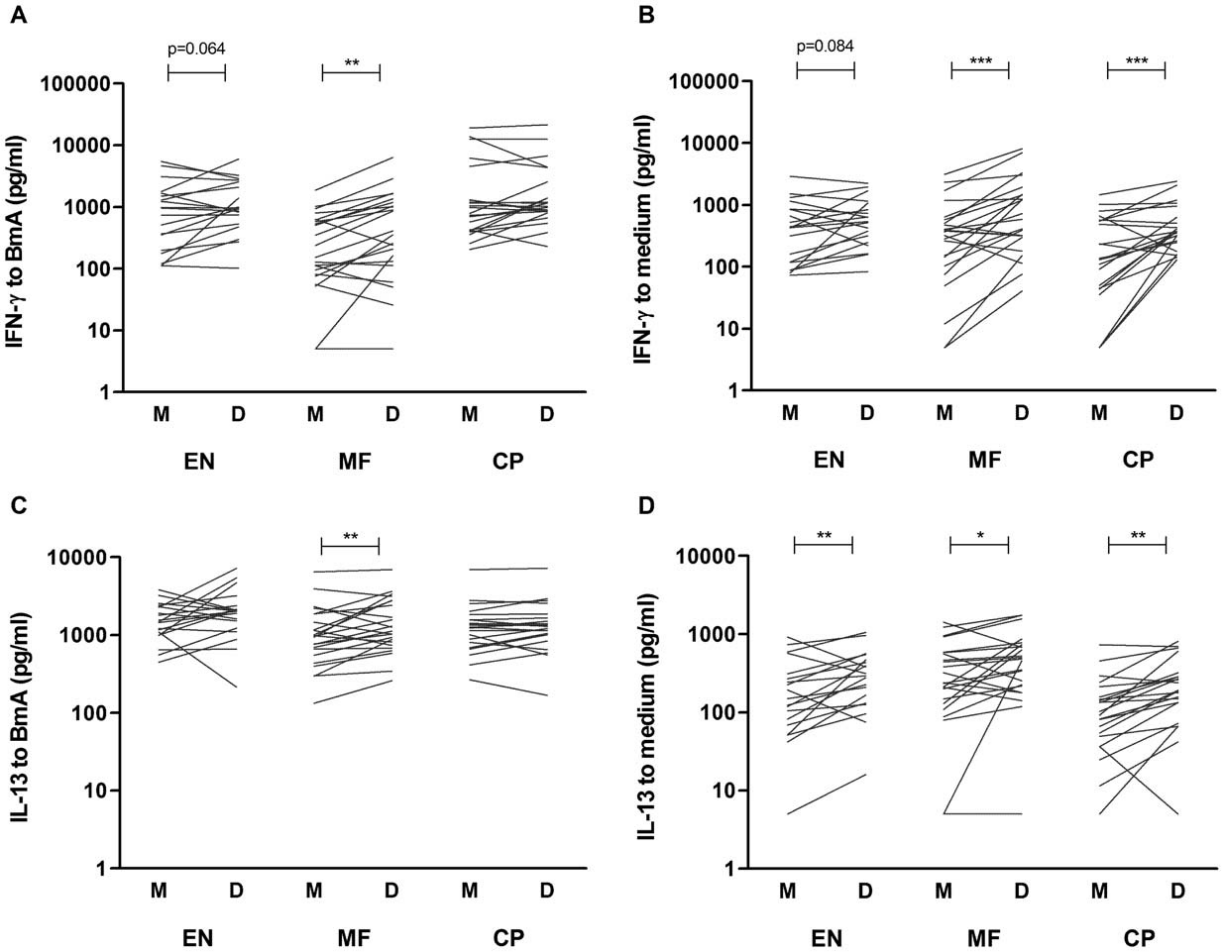
Removal of Tregs enhances filaria-specific Th1 and Th2 responses. Cytokine secretion was assessed in mock- (M) and Treg-depleted (D) PBMC cultures from EN, MF and CP individuals. IFN-γ (A–B) and IL-13 (C–D) secretion is depicted for BmA- (left panel) and unstimulated (right panel) conditions. Connecting lines represent data points of one individual, for mock- and Treg-depleted cultures; tested by paired t-test *p≤.05 **p≤.01***p≤.001, p-values between 0.05 and 0.10 are indicated.

Next, we investigated the capacity of Tregs to suppress the filaria-specific cytokines by measuring IFN-γ, IL-13, IL-17 and IL-10 in response to BmA in culture supernatants of mock- and CD4^+^CD25^hi^ - depleted PBMC. In Figure 5, unmanipulated cytokine responses to BmA and medium are shown separately. Filaria-specific IFN-γ production was significantly upregulated after removal of Tregs in the MF group only (Figure 5A; p = 0.064, p = 0.004 for EN and MF respectively). However, the IFN-γ response to BmA was weak and similar in magnitude to responses seen in medium-stimulated PBMC, which also increased after depletion of Treg in MF as well as CP (Figure 5B; p = 0.084, p = 0.0002, p = 0.001 for EN, MF and CP). With respect to IL-13, the response to BmA increased after depletion of Tregs in MF-positive individuals only (Figure 5C; p = 0.41, p = 0.008, p = 0.20 for EN, MF and CP respectively). Spontaneous IL-13 production was low compared to BmA-stimulated conditions and also increased significantly upon removal of Treg, but this was still negligible compared to levels induced by BmA (Figure 5D; p = 0.007 for EN, p = 0.038 for MF and p = 0.002 for CP). IL-17 and IL-10 responses before and after Treg depletion were comparable and unchanged in all three groups (data not shown).

## Discussion

To investigate the function of Tregs in different infection and clinical groups of human filariasis, we studied the effect of Treg depletion on *in vitro* responses to BmA using human PBMC from individuals in an area endemic for *B. timori* lymphatic filariasis in Flores, Indonesia. Our main findings were diminished T and B cell proliferation as well as lower IFN-γ, IL-17 and IL-13 production in MF-positives, but similar IL-10 secretion compared to CP and EN groups. Treg depletion resulted in antigen-specific increase of lymphocyte proliferation and IL-13 responses in the MF group only.

Since our study population was not optimally age- and sex- matched, it was necessary to adjust for age and sex in the comparisons made between the infection groups. In studies on human filariasis it is often difficult to obtain comparable patient groups. One reason for this is the pathophysiology of this disease; microfilaremia can be present in all ages but particularly in young adults, while CP is an end stage disease that develops in older age. Importantly, a recent paper demonstrated a relevant effect of age on infection-induced regulatory immune responses; intensity of infection with *Schistosoma haematobium* was positively correlated with Treg frequency in the age group 8–13 years, while the opposite was observed for the group older than 14 years [13]. Age and sex should thus be taken into account carefully when interpreting cellular immunological data.

Lymphocyte proliferation in filariasis has been studied since the 1970s and is consistently shown to be diminished in microfilaremic patients, including previous population studies by our group in Sulawesi, Indonesia [14]–[19].We have now established that the well-described T cell hyporesponsiveness can be measured by CFSE dilution assays in PBMC stimulated with BmA, and also show that in addition to T cells, B cell proliferation is considerably lower in MF-positives. Previously, it has been shown that the functional capacity of B cells, in terms of specific IgE and IgG production, was lower in MF versus CP patients [20], [21]. Here, we extend this to B cell proliferation, showing for the first time to our knowledge B cell proliferative hyporesponsiveness in microfilaremics. Interestingly, despite lower IgG found in earlier studies, the number of positive individuals for filaria-specific IgG4, an isotype shown to be associated with elevated plasma IL-10 [22], was higher in microfilaremics (data not shown). It is tempting to speculate that B cells in MF subjects that are hyporesponsive are also contributing to immune regulation by producing IL-10 and IgG4, as is suggested for venom-specific B cells from beekeepers (reviewed in [23]).

The suppressed cytokine responses in MF-positive individuals here correspond with a recent study, showing higher IFN-γ and IL-17 responses to BmA in chronic pathology patients compared to MF-positive individuals [8]. In microfilaremics IFN-γ and IL-17 production were not induced above background levels; this is also in line with the findings by Babu *et al.*, who analyzed the production of IFN-γ and the expression of IL-17 mRNA in 24 h BmA-stimulated PBMC [8]. However, Treg removal did not affect the Th1 and Th17 cytokines which may suggest that these cytokines are not regulated by Tregs. IL-13 production in response to BmA was increased after Treg depletion, however this result must be considered with caution, since medium responses were also changed. Since IL-10 levels were high in MF before as well as after Treg depletion, IL-10 derived from CD4^+^CD25^−^ T cells could be responsible for the observed decreased cytokine responses in microfilaremics, supported by two studies which showed the majority of IL-10 during filarial infection was produced by effector T cells, despite higher Tregs in the MF group [11], [24].

Contrary to our expectations, Treg depletion had little or no effect on BmA responses in the other groups, although these individuals live in a filaria-endemic area and do have filaria-specific proliferative and cytokine responses. One explanation might be that active Tregs in MF are filaria- or BmA-specific, which are only actively induced and/or expanded during patent microfilaremia. Since there are very few studies on the function of Tregs in human helminth infections, it would be interesting for future studies to determine antigen specificity and functional characteristics of the Tregs in the different study groups. Furthermore, due to limited number of available cells we were unable to determine the mechanisms by which this CD4^+^CD25^hi^ subset affects immune responses; an area that should be investigated in the future. A previous study concluded that *in vitro* blockade of CTLA-4 and PD-1 reverted suppression of *M.tuberculosis*-specific immune responses, suggesting cell-contact mediated mechanisms of suppression during microfilaremia [25].

Regarding the limitations of the current study, we were not able to evaluate whether the Treg depletion procedure has led to depletion of any other cell subsets, as a possible explanation for the reduced B cell proliferation in CP. In addition, our plan to confirm previous studies that show higher FOXP3 in *ex vivo* PBMC of MF patients failed due to a technical problem with FACS staining of FOXP3. We only had 4-days cultured PBMC that we could stain for FOXP3 and thereby we were able to show the depletion of CD25^hi^FOXP3^+^ cells. However the level of CD25 and FOXP3 in medium-cultured cells may not be fully representative for the circulating levels of Tregs. Nevertheless, although important to gather data on Treg frequencies, the primary objective of our study was to assess their functional capacity in different infection and clinical groups. It should also be mentioned that our previous study of geohelminth infection in Indonesia indicated that it was not the number but the suppressive capacity of Tregs which was altered in infected children [12].

In conclusion, we report active contribution of Tregs to modulation of T and B cell proliferation and polarized cytokine production by effector T cells in MF-positive individuals in Flores, Indonesia. Since chronic lymphedema appears to be concurrent with lack of Treg-associated suppressive capacity, further research on targeted activation of specific Tregs would be important to be able to decrease the morbidity and disabilities induced by LF.

## Supporting Information

Figure S1 **Similar spontaneous cytokine production in different disease stages.** Culture supernatants of unstimulated PBMC from EN, MF and CP subjects were assessed for IFN-γ (A), IL-17 (B), IL-13 (C) and IL-10 (D) production. Plotted values are age- and sex-adjusted means and standard errors. Mean values are not different between the three groups for all cytokines.
(TIF)

Figure S2 **Similar lymphocyte proliferative responses to filaria antigen in Treg-depleted conditions.** Divided cell populations were assessed in Treg-depleted PBMC from EN, MF and CP subjects using CFSE dilution analysis. Depicted are means and standard errors of net % divided subsets of CD4^+^CD25^+^ T cells (A) and CD19^+^ B cells (B), adjusted for age and sex. p-values between 0.05 and 0.10 are indicated.
(TIF)
